# In Vitro Cytotoxicity Effects of Zinc Oxide Nanoparticles on Spermatogonia Cells

**DOI:** 10.3390/cells9051081

**Published:** 2020-04-26

**Authors:** Ana Rita Pinho, Filipa Martins, M. Elisabete V. Costa, Ana M. R. Senos, Odete A. B. da Cruz e Silva, Maria de Lourdes Pereira, Sandra Rebelo

**Affiliations:** 1 Neuroscience and Signaling Laboratory, Institute of Biomedicine—iBiMED, University of Aveiro, 3810-193 Aveiro, Portugal; arapinho@ua.pt (A.R.P.); samartins@ua.pt (F.M.); odetecs@ua.pt (O.A.B.d.C.eS.); 2 Department of Medical Sciences, University of Aveiro, 3810-193 Aveiro, Portugal; 3 CICECO-Aveiro Institute of Materials, University of Aveiro, 3810-193 Aveiro, Portugal; elisabete.costa@ua.pt (M.E.V.C.); anamor@ua.pt (A.M.R.S.); 4 Department of Material Engineering & Ceramics, University of Aveiro, 3810-193 Aveiro, Portugal

**Keywords:** spermatogonia, ZnO nanoparticles, cytotoxicity, cell death, DNA damage, reactive oxygen species, cytoskeleton, nucleoskeleton

## Abstract

Zinc Oxide Nanoparticles (ZnO NPs) are a type of metal oxide nanoparticle with an extensive use in biomedicine. Several studies have focused on the biosafety of ZnO NPs, since their size and surface area favor entrance and accumulation in the body, which can induce toxic effects. In previous studies, ZnO NPs have been identified as a dose- and time-dependent cytotoxic inducer in testis and male germ cells. However, the consequences for the first cell stage of spermatogenesis, spermatogonia, have never been evaluated. Therefore, the aim of the present work is to evaluate in vitro the cytotoxic effects of ZnO NPs in spermatogonia cells, focusing on changes in cytoskeleton and nucleoskeleton. For that purpose, GC-1 cell line derived from mouse testes was selected as a model of spermatogenesis. These cells were treated with different doses of ZnO NPs for 6 h and 12 h. The impact of GC-1 cells exposure to ZnO NPs on cell viability, cell damage, and cytoskeleton and nucleoskeleton dynamics was assessed. Our results clearly indicate that higher concentrations of ZnO NPs have a cytotoxic effect in GC-1 cells, leading to an increase of intracellular Reactive Oxygen Species (ROS) levels, DNA damage, cytoskeleton and nucleoskeleton dynamics alterations, and consequently cell death. In conclusion, it is here reported for the first time that ZnO NPs induce cytotoxic effects, including changes in cytoskeleton and nucleoskeleton in mouse spermatogonia cells, which may compromise the progression of spermatogenesis in a time- and dose-dependent manner.

## 1. Introduction

ZnO Nanoparticles (ZnO NPs) have numerous applications due to their exceptional set of physicochemical properties, making them suitable for several biomedical applications, such as drug delivery system, bioimaging, molecular diagnostics, and cancer therapy [1,2,3]. Additionally, the use of ZnO NPs has been extended to agriculture [4], water treatment [5], industry [6], and cosmetics [7].

Considering the wide range of applications and the high human exposure to ZnO NPs, the biosafety studies of these NPs are even more imperative, given that the principal applications focus on ZnO NP capacity to generate Reactive Oxygen Species (ROS), leading to apoptosis. Nanoparticles are able to penetrate the skin, lungs, and the blood–brain barrier [8]. Several studies have demonstrated that in vivo exposure of ZnO NPs, either orally, intratracheally, or by inhalation, leads to easy accumulation in several tissues and that the rate of accumulation depends on the tissue. Liver, kidney, lung, brain, and spleen are vital organs with high levels of ZnO accumulation [9], presenting signs of cytotoxicity as a consequence of exposure and accumulation of ZnO NPs [10,11,12,13]. Furthermore, metal nanoparticles have the capacity to cross the blood–testis barrier (BTB), in part due to their size and by the generation of inflammatory response compromising the integrity of BTB [8]. Therefore, it is feasible to speculate that the ZnO NPs may also cross the BTB, inducing testicular toxicity, which is reinforced by in vivo studies that reported significant alterations in the testis after oral administration of ZnO NPs [14], even with lower doses, and by a recent study reporting an increase of Zn^2+^ accumulation in testis and epididymis [15].

ZnO NPs have been described, in recent studies, as a dose- and time-dependent cytotoxic inducer in testis and in male germ cells, leading to increased ROS production, causing DNA damage in some spermatocytes [16,17,18,19] and ultimately apoptosis [18], cell cycle arrest, and downregulation of BTB protein levels, compromising BTB integrity [19]. These cellular effects have repercussions on histological integrity, causing disorganization and detachment of the germ cell layer and vacuolation of germ cells [20,21,22]. In addition, an increase of sperm abnormalities and a decrease in sperm counts were observed after ZnO NPs exposure [10,16,17,18,21,23]. Such a phenotype is consistent with the pattern of spermatogenesis arrest. Further, the impact of ZnO NPs on the male reproductive system was recently reviewed [14]. Overall, it is important to clarify the consequences of ZnO NPs exposure in the first cell stage of spermatogenesis, spermatogonia, and how these compromises spermatogenesis progression.

Previous studies have indicated that nanomaterials can negatively affect male germ cells in different ways. A recent study identified ZnO NPs as a cause of an acute cytoskeletal collapse as a result of intracellular dissolution of ZnO, causing ROS accumulation which culminates with apoptotic cell death [24,25,26]. Besides causing cytoskeleton alterations, ZnO NPs were also reported to be responsible for nuclear enlargement [25]. Further studies demonstrated that these reported nuclear envelope (NE) alterations were not a direct consequence of NPs but a consequence of cytoskeleton disruption, leading to nuclear membrane disruption [8,25]. The linker of the nucleoskeleton and cytoskeleton (LINC) complex crosses the NE and has crucial functions in controlling the nuclear and cytoplasmic activities through the organization of nuclear and cytoskeletal characteristics. LINC complexes are formed by interaction of Klarsicht; ANC-1; Syne Homology (KASH); and Sad1p, UNC-84 (SUN) domain proteins, providing a physical link between the cytoskeleton and the nuclear interior [27]. Further, there is growing evidence that A-type lamins and NE proteins play a critical role in responding to mechanical cues from the extracellular matrix by adjusting the cytoskeletal and nuclear stiffness [28].

Since the ZnO NPs adverse effects on male reproductive systems are not fully elucidated and the consequences of exposure to ZnO NPs in spermatogonia have never been studied, the present work aims to investigate in vitro the cytotoxic effects of ZnO NPs using spermatogonia cells as model (GC-1 cell line). GC-1 cells are a mouse-derived spermatogonia cell line, described as an intermediate spermatogenic cell type between type B spermatogonia and primary spermatocytes, representing an attractive cell model to explore the effects of ZnO NP exposure in a premeiotic spermatogenesis stage. GC-1 cells were incubated with different doses of ZnO NPs for 6 h and 12 h. Cell viability at the metabolic level and cell integrity were evaluated. The intracellular ROS levels, DNA damage by monitoring the phosphorylation of γ-H2AX (S139), and cell death rate were also assessed. To evaluate possible alterations in the cytoskeleton of spermatogonia cells, protein levels of α-tubulin-acetylated and β-tubulin, structural proteins of microtubules, were quantified by immunoblotting and immunocytochemistry. β-actin and F-actin, constituents of microfilaments, were assessed by immunoblotting and immunocytochemistry, respectively. Evaluation of the LINC complex, namely SUN1 (containing the SUN domain) and Nesprin-1 (containing the KASH domain) proteins, and other highly relevant NE proteins for spermatogenesis [29,30], lamin A/C and lamina-associated polypeptide 1 (LAP1), was also achieved by immunocytochemistry.

## 2. Materials and Methods

### 2.1. ZnO NPs

A commercial ZnO nanopowder (<100 nm) (Sigma-Aldrich, Saint Louis, MO, USA) was used. The structure of the powder was characterized by X-ray diffraction (XRD) using a Panalytical X’Pert Pro Diffractometer (PANalytical B.V., Almelo, The Netherlands) with Cu Kα1 radiation (λCu = 0.154056 nm). The XRD patterns were recorded in the range of 10–80° of 2q values, with a 0.02° step and time per step of 3 s. The powder specific surface area was accessed by gas adsorption (BET isotherm) in a Micromeritics-Gemini V2380 surface area analyser (Micromeritics, Norcross, GA, USA). Scanning electron microscopy (SEM) was performed to analyse the powder morphology using a Hitachi SU-70 Scanning Electron Microscope (Hitachi High-Tech, Tokyo, Japan). The particle surface charge was also accessed by measuring the Zeta potential of ZnO aqueous suspensions at different pHs in Coulter Delsa 440 SX equipment (Beckman Coulter, Indianapolis, IN, USA).

### 2.2. Antibodies

The primary antibodies used for immunoblotting were mouse monoclonal γ-H2AX (S139) (Millipore, Darmstadt, Germany; 1:500), mouse monoclonal β-tubulin (Invitrogen, Thermo Fisher Scientific, Waltham, MA, USA; 1:1000), mouse monoclonal acetylated α-tubulin (Sigma-Aldrich, Saint Louis, MO, USA; 1:2000), and mouse monoclonal β-actin (Novus Biologicals, Centennial, CO, USA; 1:5000). The secondary antibody used was anti-mouse horseradish peroxidase-linked antibody (Cell Signalling Technology, Danvers, MA, USA; 1:10,000) for ECL detection.

For immunocytochemistry analysis, the primary antibodies used were mouse monoclonal α-tubulin-acetylated (Sigma-Aldrich, Saint Louis, MO, USA; 1:250), mouse monoclonal β-tubulin (Life Technologies, Carlsbad, CA, USA; 1:500), mouse monoclonal nesprin-1 (Developmental Studies Hybridoma Bank, Iowa City, IA, USA; 1.5 µg/mL), mouse monoclonal lamin A/C (Sigma-Aldrich, Saint Louis, MO, USA; 1:250), rabbit polyclonal LAP1 [31] (Goodchild and Dauer, 1:40,000), and rabbit SUN1 (kindly provided by our collaborator; 1:500). The secondary antibodies goat anti-mouse IgG Alexa Fluor 488 (Invitrogen, Thermo Fisher Scientific, Waltham, MA, USA; 1:300) and goat anti-rabbit IgG Alexa Fluor 594 (Invitrogen, Thermo Fisher Scientific, Waltham, MA, USA; 1:300) were used.

### 2.3. Cell Culture and Cell Exposure to ZnO NPs

An immortalized cell line was used to study the effects of exposure to ZnO NPs. GC-1 spg (ATCC^®^ CRL2053™, Manassas, VA, USA) cells are derived from postnatal day-10 mouse testis and present characteristics of a premeiotic stage between type B spermatogonia and primary spermatocytes. Given these GC-1 cell characteristics, they are a cell model with scientific relevance for male reproductive system studies as indicated by their use in relevant studies about the male reproductive system [32,33]. GC-1 cells were grown in Dulbecco’s Modified Eagle’s Medium (DMEM, bioWest, Riverside, MO, USA) supplemented with 10% (*v*/*v*) of Fetal Bovine Serum (FBS; bioWest) and 1% (*v*/*v*) penicillin-streptomycin solution (Pen Strep; bioWest). Cell culture was maintained at 37 °C and 5% CO_2_ atmosphere. Cells were seeded and cultured for 16 h prior to being washed with phosphate-buffered saline (PBS; pH 7.4) and incubated in a fresh complete growth medium containing the different concentrations of ZnO NPs (0, 1, 5, 8, 10, and 20 µg/mL) for 6 and 12 h. The selection of both concentration and duration of the experiments was achieved by a comprehensive and critical analysis of previous studies performed in the area followed by preliminary studies carried out in GC-1 cells. The objective was the selection a set of concentrations ranging low (1, 5, and 8 µM) and high ZnO NP doses (10 and 20 µM). Further short- (6 h) and long-term (12 h) exposures were selected. ZnO NP suspensions with different concentrations were prepared diluting a stock suspension of higher concentration in complete culture medium, ensuring the addition of same volume of ZnO NPs at each experimental condition. ZnO NPs were sterilized by UV radiation for 20 min and sonicated before use in cell culture.

### 2.4. Cell Viability Assays

Cell viability analysis was performed using three different approaches: the resazurin assay, the trypan blue exclusion method, and flow cytometry analysis of Annexin V/propidium iodide (PI). For all approaches, GC-1 cells were seeded in 6-well plates (Orange scientific) at a density of 2.5 × 10^5^/well and were treated with fresh medium containing different concentrations of ZnO NPs for 6 and 12 h, as described above. For the resazurin assay, during exposure to ZnO NPs, cells were incubated 4 h with 10% Resazurin Sodium Salt (Sigma-Aldrich) in complete DMEM medium. The culture medium was collected at the end of incubation times (6 and 12 h), transferred to a 96-well plate, and subsequently analysed. The resazurin reduction was measured spectrophotometrically at 570 and 600 nm (Infinite M200 PRO, Tecan, Switzerland). The OD 570/OD 600 nm ratio was calculated for each condition and presented as arbitrary units over untreated cells per time point. The cell viability of the control condition received the value of 100. For trypan blue analysis at the end of the incubation periods (6 and 12 h), GC-1 cells were collected and resuspended in fresh DMEM medium. The number of viable and dead cells was counted using a 0.4% trypan blue dye solution and an haemocytometer. The values, similar to the resazurin assay, are expressed as arbitrary units, and the cell viability of the control condition was given the value of 100.

The cell death was measured by flow cytometry analysis of Annexin V/PI. For these assays, GC-1 cells were collected 6 h and 12 h after exposure to 0, 5, 10, and 20 µg/mL of ZnO NPs. The cell suspension was centrifuged at 1000 rpm for 3 min at 4 °C, and the cell pellet was resuspended in binding buffer (1× PBS with Ca^2+^ at a concentration of 0.33 g/L). Further, a centrifugation at 1000 rpm for 3 min at 4 °C was performed, the supernatant was discarded, and the cell pellet was resuspended in binding buffer with conjugated recombinant Annexin V (Annexin V-APC, Immuno Tools, Friesoythe, Germany). Cells were exposed to Annexin V-APC for 15 min in the dark. Finally, propidium iodide staining solution (PI; BD Pharmingen™, San Jose, CA, USA) (100 µg/mL) was added, and cytometry analyses were performed using a BD Accurati^TM^ C6 (BD Biosciences^®^, San Jose, CA, USA). Proper negative and positive controls were also analyzed. For the negative control, cells were incubated without annexin V-APC or PI. For positive control, cells were treated with 100 µM of hydrogen peroxide (H_2_O_2_) for 2 h and stained with annexin V-APC and PI. The results were analysed using a BD AccuratiTM C6 Software (BD Biosciences^®^).

### 2.5. Evaluation of Intracellular ROS Levels

The intracellular ROS levels were detected using the Total ROS Detection kit (ENZO Life Sciences, Lausen, Switzerland), which contains a 2,7-dichlorodihydrofluorescein diacetate (DCFH-DA) probe. DCFH-DA diffuses through the cellular membrane and accumulates in cytoplasm. Intracellular esterases first hydrolyse the non-fluorescent lipophilic probe DCFH-DA to non-fluorescent 2,7-dichlorodihydrofluorescein (DCFH), which is then oxidized by reactive species and originates 2,7-dichlorofluorescein (DCF), a fluorescent compound that might be quantified [34,35,36,37]. GC-1 cells were seeded in 96-well plates at a density of 1 × 10^4^ cells/well. At the end of incubation with ZnO NPs (6 and 12 h), the fluorescein fluorescence intensity, with fluorescence excitation and emission at 488 and 520, respectively, was measured using a fluorescence microplate reader (Infinite M200 PRO, Tecan) without removing the detection mix. For the negative control, cells were incubated with a ROS inhibitor—*N*-acetyl-l-cysteine (NAC). For the positive control, cells were exposed to the ROS inducer—pyocyanin. The procedure was done following the manufacturer instructions for total ROS Detection kit.

### 2.6. SDS-PAGE and Immunoblotting

GC-1 were seeded in 6-well plates at a density of 2.5 × 10^5^/well for 16 h. Cell lysates from GC-1 cells incubated for 6 and 12 h with different concentrations of ZnO NPs were collected in a 1% SDS solution, and the total protein content was quantified using the Pierce’s bicinchoninic acid (BCA) protein assay kit (Thermo Fisher Scientific). Samples were further separated into a 5–20% gradient SDS-PAGE, and immunoblotting analysis was performed. The total amount of protein content in the nitrocellulose membrane (GE Healthcare, Chicago, IL, USA) was detected by reversible Ponceau S staining (Sigma-Aldrich) and then scanned on a GS-800 calibrated imaging densitometer (Bio-Rad, San Jose, CA, USA). The immunoblotting analysis started with membrane blocking with 5% BSA in 1× TBST. The incubations of primary antibodies were performed overnight at 4 °C. The anti-mouse horseradish peroxidase-conjugated secondary antibodies were incubated for 2 h at room temperature. To detect protein bands, the ECL™ Select Western Blotting detection reagent (GE Healthcare was used. Immunoblots were scanned and quantified (GS-800™ Calibrated Densitometer and Quantity One densitometry software, Bio-Rad), and the data were normalized for to the respective Ponceau loading control as previously described by Santos et al. [38].

### 2.7. Immunocytochemistry and Confocal Microscopy Analysis

Cells were seeded in 6-well plates at 2 × 10^5^ cells/well. After 16 h, GC-1 cells were treated with fresh media containing 0 and 20 µg/mL of ZnO NPs for 6 h and 12 h. Cells were fixed in 3.7% paraformaldehyde and permeabilized with 0.2% Triton X-100 in 1× PBS. Additionally, cells were blocked with 3% BSA in 1× PBS for 1 h. Cells were incubated with the primary antibodies for 2 h, followed by the secondary antibody for 1 h. To stain filamentous actin (F-actin), Alexa Fluor^®^ 568 Phalloidin (Molecular probes, Invitrogen, Thermo Fisher Scientific, Waltham, MA, USA; 1:500) was added for 1 h and as previously described [39]. Finally, the coverslips were mounted on a microscope slide with 4’,6-diamidino-2-phenylindole (DAPI)-containing VECTASHIELD^®^ Mounting media (Vector Laboratories, Peterborough, UK). Preparations were visualized using an LSM880 confocal microscope (Zeiss) and a 63×/1.4 oil immersion objective. Microphotographs were acquired in a sole section in the Z-axis and represent a mean of 8 scans. Fluorescence intensity analyses were performed using ImageJ software (U.S. National Institutes of Health). The values are presented as relative fluorescence intensity and, for the fluorescence intensity of each protein at control condition, was given the value of 100. The fluorescence intensity of each condition was accordingly calculated in comparison to control.

### 2.8. Statistical Analysis

Statistical analysis was performed using the GraphPad Prism 6.0 software using two-way ANOVA followed by Dunnett’s multiple comparisons test with a statistical confidence coefficient of 95% for comparisons between time points and concentrations. One-way ANOVA was used, followed by the Dunnett’s test, with a statistical confidence coefficient of 95% for comparisons between concentrations per time point. All data were expressed as mean ± standard error of the mean. The *p*-values for each statistical test applied (* for two-way ANOVA test; ^#^ for one-way ANOVA test) are indicated in the results and in the legends of figures.

## 3. Results 

### 3.1. Characterization of ZnO NPs

**Figure 1:**
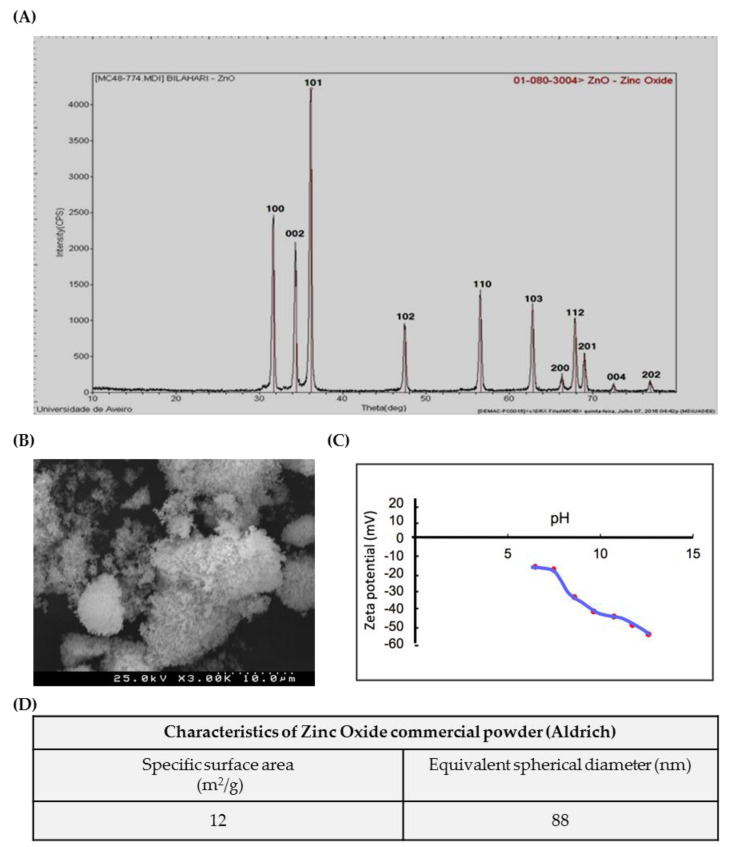
Characterization of ZnO nanoparticles (NPs): Characterization of commercial ZnO powder: (**A**) XRD; (**B**) SEM image; (**C**) Zeta potential curve, and (**D**) table summarizing the morphological characteristics. XRD—X-ray diffraction; SEM—Scanning electron microscopy.

ZnO nanoparticles used in the present study were characterized according to a set of tests (Figure 1). Figure 1A shows the XRD Analysis pattern of ZnO nanoparticles. All the diffraction peaks correspond to the characteristic hexagonal wurtzite structure of zinc oxide, spatial group P63mc, and cell parameters a = 3.252 A^0^ and c = 5.214 A^0^, as specified in the card number 01-089-1397 (Committee on Powder Diffraction Standards (JCPDS), International Centre for Diffraction Data). The SEM microphotograph in Figure 1B reveals that the powder exhibits an agglomeration state of very fine particles, with nanometric size. Accordingly, the high value of specific surface area, determined based on BET isotherm, S_BET_ = 12 m^2^/g (Figure 1D), corresponds to an average equivalent spherical diameter in the nanometric range G_BET_ = 88 nm. Figure 1C shows the Zeta potential variation of ZnO aqueous suspensions with increasing pH values, from 6 to 12; it can be observed that the Zeta potential is negative, starting in −15 mV at pH = 6 and becoming more negative with increasing pH (–55 mV at pH = 12).

### 3.2. ZnO NPs Reduce the Viability of GC-1 Cells in a Dose- and Time-Dependent Manner

**Figure 2:**
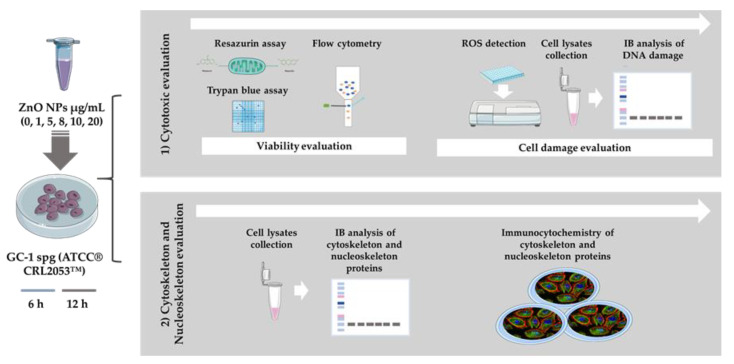
Schematic representation of experimental workflow: Methods used for evaluation of the cytotoxic effects of ZnO NPs in the GC-1 spg cell line (ATCC^®^ CRL2053™). The study was divided in two stages. Initially, cytotoxicity was evaluated by cell viability and cell damage analysis (oxidative and DNA damage), and then, the alterations of cytoskeleton and nucleoskeleton were also pursued. IB—Immunoblotting; ZnO NPs—Zinc Oxide Nanoparticles.

ZnO NPs has been described as a cytotoxic inducer affecting different cell types, including cells from the male reproductive system, namely spermatocytes [19], spermatozoa [17], Sertoli cells [18,19], and Leydig cells [18]. However, spermatogonia cells had never been evaluated before. Of note, the periods of incubation selected for this study were considerably short and the set of concentrations of ZnO NPs tested were presumably low (see the Material and Methods section). The objective was to determine if these short exposure periods to different ZnO NP amounts induced alterations in spermatogonia cells. For this purpose, different approaches summarized in Figure 2 were used. 

**Figure 3:**
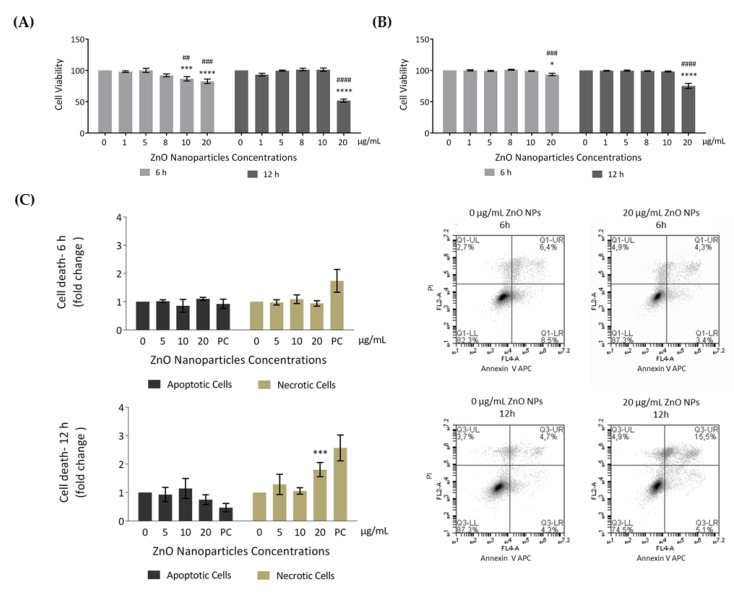
Evaluation of cell viability induced by ZnO NPs in GC-1 spg cells: (**A**) Cell viability was assessed using the resazurin assay. Results from the viability analysis of GC-1 cells after exposure for 6 h and 12 h to different ZnO NP concentrations: The viability for each condition is presented as mean ± SEM of seven independent experiments. Values are expressed as arbitrary units, and the cell viability of the control condition was given a value of 100. (**B**) Cell viability was assessed using the trypan blue exclusion method. Trypan blue analysis of GC-1 cells after exposure for 6 h and 12 h to different ZnO NPs concentrations: The viability for each condition is presented as mean ± SEM of six independent experiments. Values are expressed as arbitrary units, and the cell viability of the control condition was given a value of 100. (**C**) Cell viability was assessed by flow cytometry analysis of Annexin V/ propidium iodide (PI). Flow cytometry analysis of Annexin V-APC and PI staining and of membrane and DNA markers, respectively, in the GC-1 cell line after exposure to 0, 5, 10, and 20 µg/mL of ZnO NPs for 6 h and 12 h. Positive control was performed using H_2_O_2_. The fold change in controls (cells without ZnO NPs) of apoptotic and necrotic cells was plotted as mean ± SEM of four independent experiments, for each condition. * For comparisons between concentrations and time points, two-way ANOVA was used. # For comparisons between concentrations, one-way ANOVA was used. *^/#^ *p* < 0.05. **^/##^ *p* < 0.01. ***^/###^ *p* ≤ 0.001. ****^/####^ *p* < 0.0001. PI—Propidium Iodide. PC—Positive Control.

In order to evaluate the effects of ZnO NPs in cellular metabolic activity (resazurin assay) and cell membrane integrity (trypan blue exclusion method), GC-1 cells were incubated with different concentrations of ZnO NPs for 6 and 12 h. According to results from resazurin assay (Figure 3A), the cell viability significantly decreased after 6 h of incubation with 10 µg/mL (* *p* ≤ 0.001; ^#^ *p* < 0.01) and 20 µg/mL (* *p* < 0.0001; ^#^ *p* ≤ 0.001), corresponding to cell viability decreases of 13% and 17%, respectively. Upon 12 h of ZnO NP exposure, cell viability was only altered with 20 µg/mL of ZnO NPs (*^/#^ *p* < 0.0001), decreasing 48% when compared with control (ZnO NP unexposed cells for 12 h). Moreover, using the second cell viability approach (trypan blue), the results were quite similar (Figure 3B). However, cell viability only decreased significantly when using the higher ZnO NP concentration (20 µg/mL) for either 6 h (* *p* < 0.05; ^#^ *p* ≤ 0.001) or 12 h (*^/#^ *p* < 0.0001).

To further analyze cell death, an Annexin V/PI staining assay was performed to discriminate between viable, apoptotic, and necrotic cells through differences in plasma membrane integrity and permeability. Both apoptotic and necrotic cells are stained with Annexin V, but they are distinguished by co-staining with PI given that, during necrosis, the cell membrane integrity is lost and PI can cross the cell membrane [40,41]. GC-1 cells were treated with 0, 5, 10, and 20 µg/mL of ZnO NPs for 6 h and 12 h, and the apoptotic and necrotic cells were monitored by flow cytometry. The results indicated a significant increase in number of necrotic cells at an exposure dose of 20 µg/mL ZnO NPs for 12 h (* *p* ≤ 0.001) (Figure 3C). Thus, high concentrations of ZnO NPs and a longer exposure period can induce cell death of GC-1 cells. Given the cell viability alterations observed, characterization of the type of damage induced by ZnO NPs in GC-1 cells was performed.

### 3.3. Evaluation of Cell Damage Induced by ZnO NPs

In order to evaluate the type of cell damage observed upon exposure to ZnO NPs, the generation of ROS intracellular levels (oxidative damage) and the occurrence of DNA damage were monitored.

#### 3.3.1. ROS Intracellular Levels Increase (Oxidative Damage)

**Figure 4:**
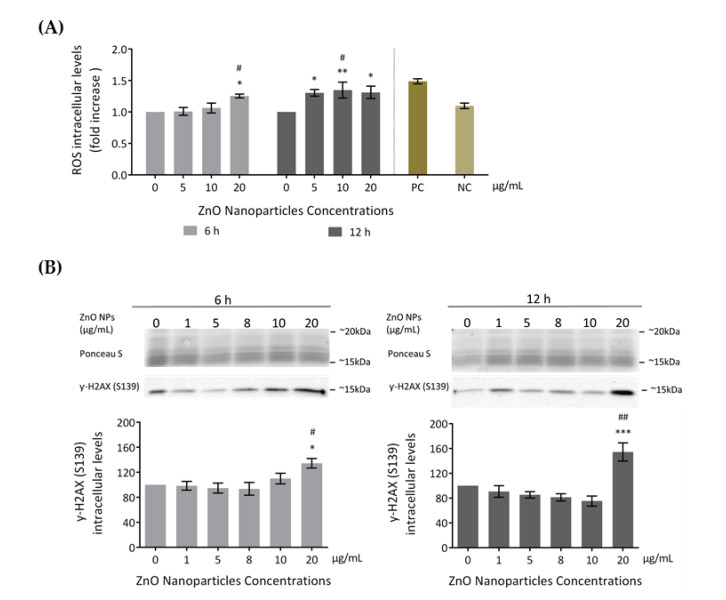
Cell damage induced by ZnO NPs: (**A**) Oxidative damage. Reactive Oxygen Species (ROS) intracellular level detection using the Total ROS Detection kit (ENZO Life Sciences) after the exposure of GC-1 cells to different concentrations of ZnO NPs. Positive and negative controls were performed using pyocyanin and *N*-acetyl-l-cysteine (NAC), respectively. The ROS levels were plotted as fold increase over the control (cells without ZnO NPs) for both 6 h and 12 h. The values for each condition were presented as a mean ± SEM of four independent experiments. (**B**) DNA damage: Analysis of γ-H2AX (Ser 139), a marker of DNA damage, by immunoblotting in GC-1 cells treated with different concentrations of ZnO NPs for 6 and 12 h. Protein levels are presented as a fold increase (%) over controls, which was plotted as mean ± SEM of four independent experiments. * For comparisons between concentrations and time points, two-way ANOVA was used. # For comparisons between concentrations, one-way ANOVA was used. *^/#^ *p* < 0.05. **^/##^ *p* < 0.01. *** *p* ≤ 0.001. NAC—*N*-acetyl-l-cysteine. NC—Negative Control. PC—Positive Control. ROS—Reactive Oxygen Species.

Several studies reported increased ROS production as the cause of cytotoxicity upon exposure to ZnO NPs [18,19]. Therefore, to evaluate if ZnO NPs induce ROS level alterations in GC-1 cells, ROS intracellular levels were assessed using the total ROS detection kit after incubation with 0, 5, 10, and 20 μg/mL of ZnO NPs for 6 and 12 h (Figure 4A). GC-1 exposure to ZnO NPs (20 μg/mL) for 6 h significantly increased ROS production compared to the control group (*^/#^ *p* < 0.05). At 12 h, a significant alteration of ROS production levels was detected in cells exposed to 5 (* *p* < 0.05), 10 (* *p* < 0.01, ^#^ *p* < 0.05), and 20 μg/mL (* *p* < 0.05) of ZnO NPs. These results suggest that high ZnO NP concentrations and longer exposure times to these NPs were associated with higher generation of ROS levels.

#### 3.3.2. Occurrence of DNA Damage

Several studies indicated that increased ROS is the cause of DNA damage in cell lines exposed to ZnO NPs [18,19,42]. In the present study, DNA damage was assessed through detection of γ-H2AX phosphorylation (S139) intracellular levels by immunoblotting (Figure 4B). γ-H2AX is a marker of DNA double-strand breaks [43]. GC-1 cells exposed to different doses of ZnO NPs for 6 and 12 h showed an increase of γ-H2AX (S139) levels in a dose- and time-dependent manner. This increase was significant at 20 µg/mL after 6 h of exposure (*^/#^ *p* < 0.05) and highly significant at 20 µg/mL after 12 h of treatment (* *p* ≤ 0.001; ^#^ *p* < 0.01). Therefore, the exposure to the highest ZnO NP concentration tested is a genotoxic factor by inducing DNA damage in the GC-1 cell line. Ponceau S staining was used as a loading control, as previously described.

### 3.4. ZnO NPs Influence the Cytoskeleton in GC-1 Cells

**Figure 5:**
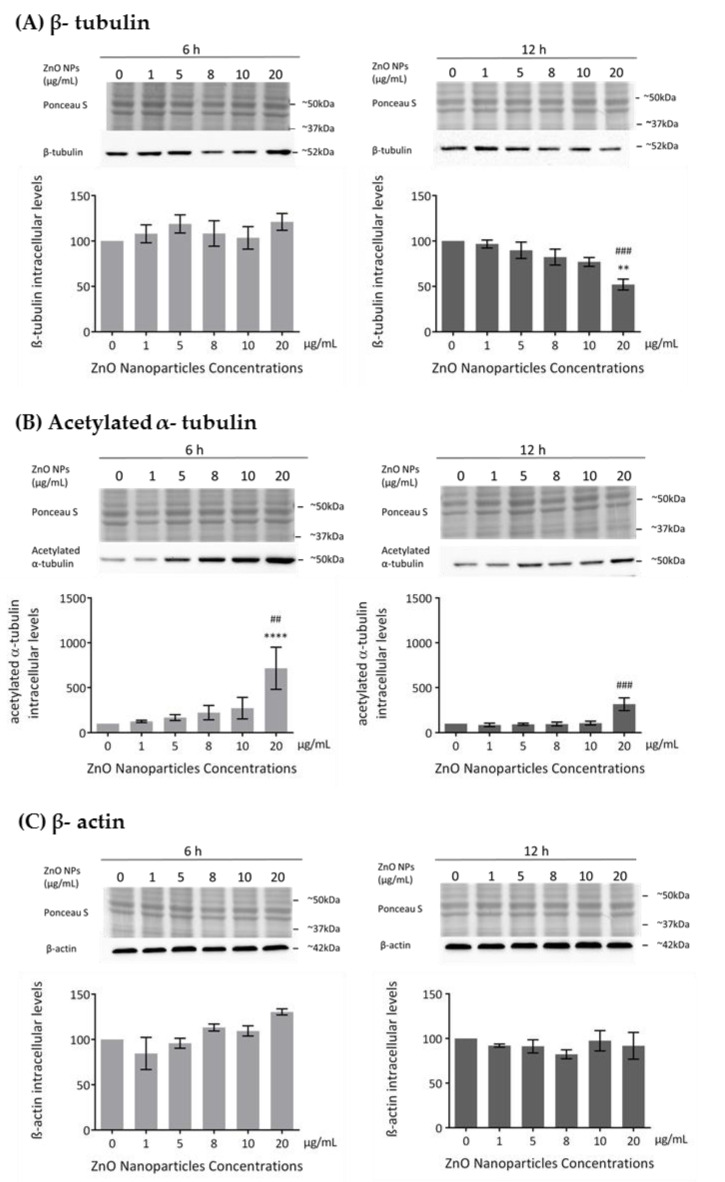
Influence of ZnO NPs in cytoskeleton structure and dynamics of GC-1 spg cells: (**A**) Quantification of β-tubulin, (**B**) acetylated α-tubulin, (**C**) β-actin protein levels in GC-1 cells by immunoblotting analysis. Cells were exposed to ZnO NPs for 6 h and 12 h. Protein levels are presented as a fold change (%) over controls, which was plotted as mean ± SEM of three or four independent experiments. * For comparisons between concentrations and time points, two-way ANOVA was used. ^#^ For comparisons between concentrations, one-way ANOVA was used. **^/##^ *p* < 0.01. ^###^ *p* ≤ 0.001. **** *p* < 0.0001.

The cytoskeleton is a very important component in cell structure and metabolism. As reported in this study, ZnO NPs decrease cell metabolism and membrane integrity in a dose-dependent manner, which is in agreement with the increase in cell death observed and which is associated with severe morphological changes in proteins of cytoskeleton, such as tubulin and actin [44]. Previous studies described the ZnO NPs as a cause of cytoskeletal dynamic alterations in different types of cells but not in spermatogonia cells [24,25,26]. Therefore, the evaluation of the cytoskeleton integrity and dynamics was monitored in GC-1 cells upon their exposure to different concentrations of ZnO NPs for 6 and 12 h. Microtubule changes were assessed by analyzing the levels of β-tubulin and acetylated α-tubulin and alterations in the filaments through the levels of β-actin and F-actin. The β-tubulin levels decreased in a ZnO NP dose-dependent manner after 12 h of NP exposure (Figure 5A). This decrease was only significant with the highest concentration of ZnO NPs tested (* *p* ≤ 0.01; ^#^ *p* ≤ 0.001). However, no alterations of β-tubulin comparative to control were observed after 6 h of GC-1 exposure to ZnO NPs (Figure 5A). Significant alterations on acetylated α-tubulin levels (a marker of microtubules stability) after GC-1 exposure to ZnO NPs were observed. The acetylated α-tubulin levels were significantly increased when GC-1 cells were incubated with 20 µg/mL of ZnO NPs for both periods of incubation (Figure 5B): at 6 h (* *p* < 0.0001; ^#^ *p* < 0.01) and at 12 h (^#^ *p* ≤ 0.001). No significant alterations in the β-actin intracellular levels were verified by immunoblot analysis (Figure 5C). However, a slight progressive increase dependent of dose was observed at 6 h of incubation. Ponceau S staining was used as a loading control, as previously described.

**Figure 6:**
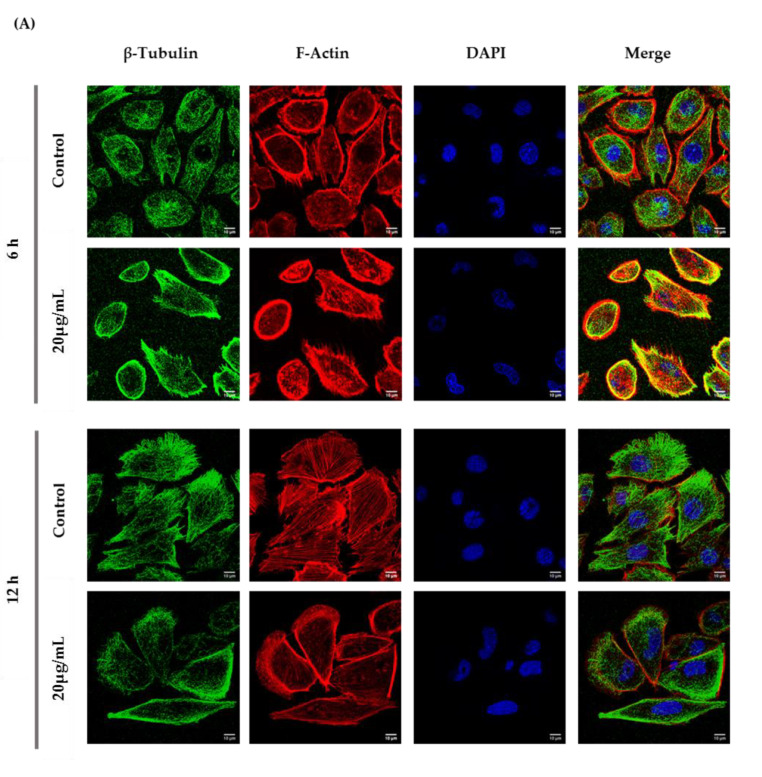

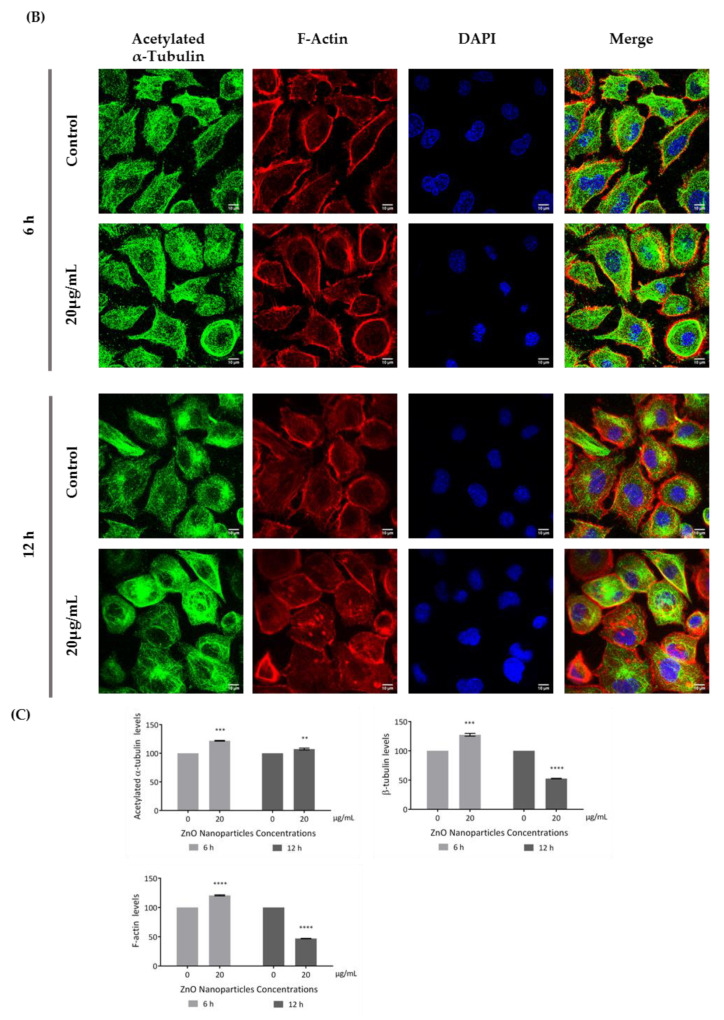

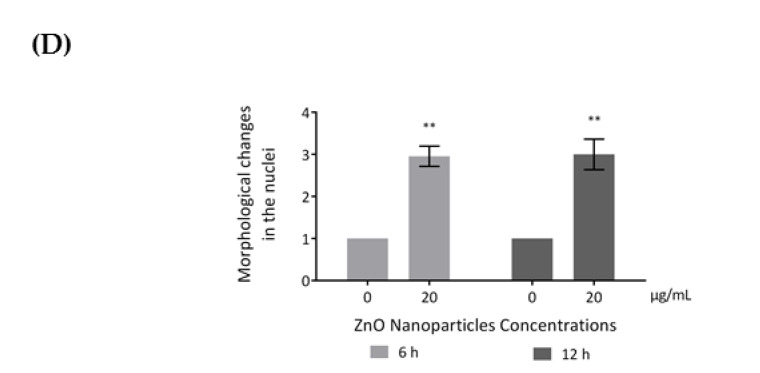
Influence of ZnO NPs in cytoskeleton structure and in nuclear morphology of GC-1 spg cells by immunocytochemistry: Immunocytochemistry images of (**A**) β-tubulin, (**B**) acetylated α-tubulin, and (**A**,**B**) F-actin; (**C**) relative fluorescence intensity quantification of α-tubulin, β-tubulin, and F-actin protein levels; and (**D**) morphological analysis of nuclei in GC-1 spg cell line. The cells were exposed to 0 and 20 µg/mL ZnO NPs for 6 h and 12 h. The protein levels are presented as a fold change (%) over controls, which was plotted as mean ± SEM of three independent experiments. Each experiment was obtained by analyzing at least 30 cells per condition. The percentage of cells with nuclear morphological changes is shown as a fold change, which was plotted as mean ± SEM of four independent experiments. Each experiment was obtained by analyzing at least 60 nuclei per condition ** *p* < 0.01. *** *p* ≤ 0.001. **** *p* < 0.0001. * For comparisons between concentrations and time points, two-way ANOVA was used.

According to the immunocytochemistry analysis, microtubule and microfilament protein levels changed after GC-1 cell exposure to 20 µg/mL of ZnO NPs for 6 and 12 h (Figure 6). The actin dynamics of GC-1 cells was monitored by F-actin, a specific marker of actin polymerization. β-tubulin (* *p* ≤ 0.001) and F-actin (* *p* < 0.0001) increased significantly after 6 h of ZnO NP exposure to 20 µg/mL. However, after 12 h, GC-1 cells presented a significant reduction of β-tubulin (* *p* < 0.0001) and F-actin (* *p* < 0.0001) levels with the highest concentration tested relative to control cells. Moreover, at 6 h of ZnO NP treatment, GC-1 cells showed an increase of acetylated α-tubulin levels (* *p* ≤ 0.001) with 20 µg/mL. Similarly, after 12 h of ZnO NP exposure, acetylated α-tubulin levels also increased significantly (* *p* < 0.01) with the same concentration.

### 3.5. ZnO NPs Influence the Nucleoskeleton Integrity in GC-1 Cells

**Figure 7:**
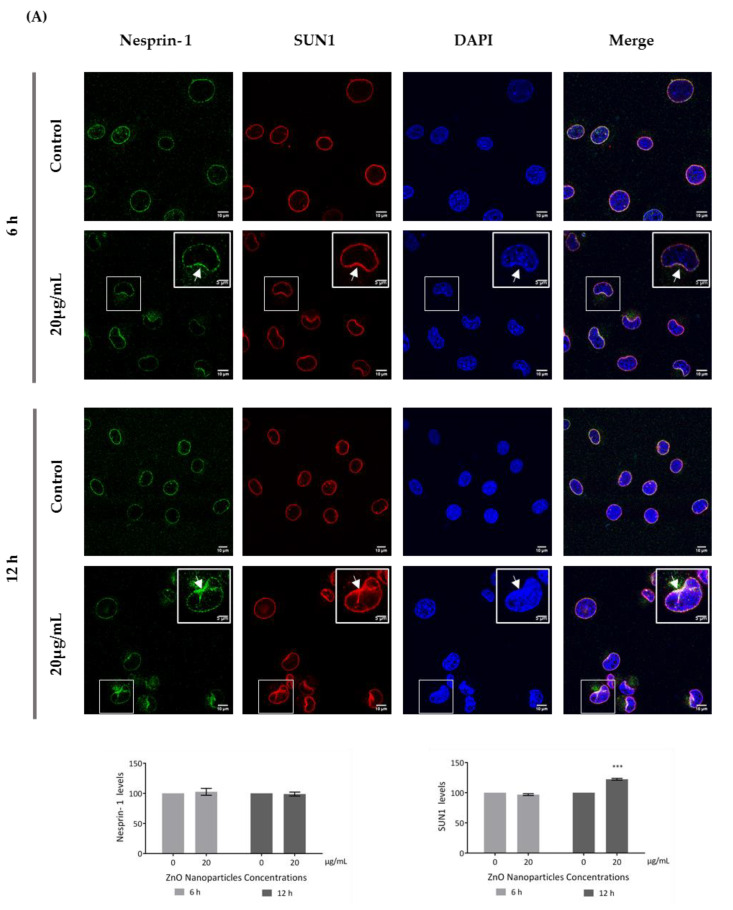

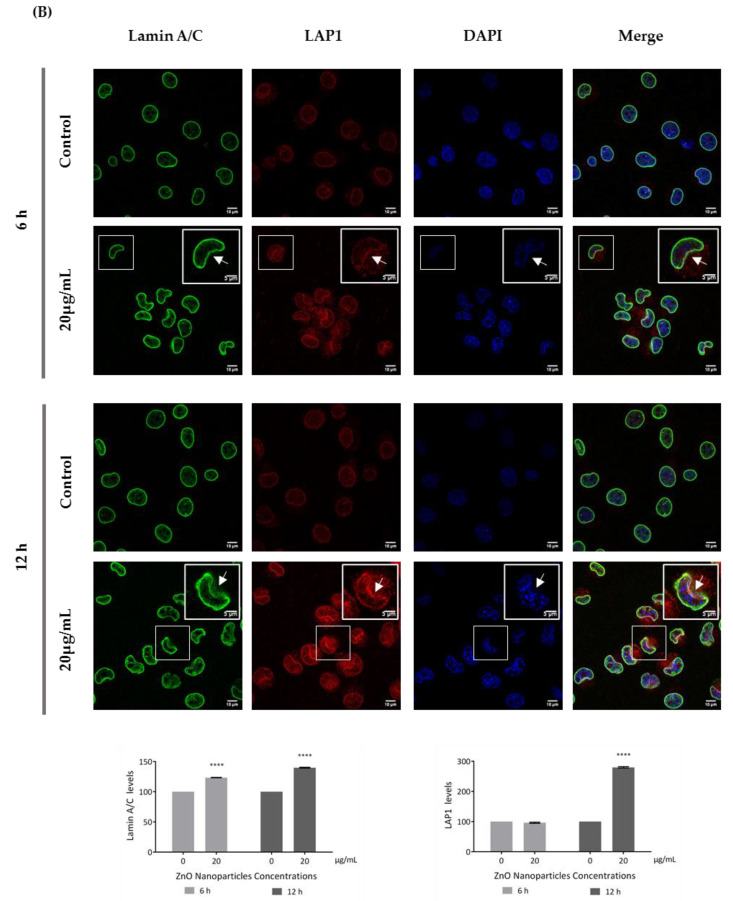
Influence of ZnO NPs in nucleoskeleton structure of GC-1 spg cell line by immunocytochemistry: Immunocytochemistry images of (**A**) nesprin-1 and SUN1, and (**B**) lamin A/C and LAP1 in the GC-1 spg cell line and the respective relative fluorescence intensity quantification. Cells were exposed to 0 and 20 µg/mL ZnO NPs for 6 h and 12 h. Areas of nucleus confinement are evident (arrows). Protein levels are presented as a fold change (%) over controls, which was plotted as mean ± SEM of three independent experiments. Each experiment was obtained by analyzing at least 40 cells per condition. *** *p* ≤ 0.001. **** *p* < 0.0001.

Recent reports have stated that ZnO NPs can induce cytoskeleton alterations, thereby destroying the dynamic nature of the cell. These changes in cytoskeleton can influence the nucleus structure [24]. According to immunocytochemistry images of the DAPI-labelled nucleus, significant morphological nuclear changes were observed after exposure of GC-1 cells to higher levels of ZnO NPs (Figure 6D). The number of GC-1 nuclei with asymmetric constriction characterized by a nuclear reniform, a multilobulated, or a blebbed shape increased in the presence of ZnO NPs (Figure 6 and Figure 7).

This is an interesting result that was, for the first time, subsequently pursued by the evaluation of the effects of ZnO NPs on the nucleoskeleton (nuclear lamina, and NE proteins) in spermatogonia cells. SUN1, nesprin 1, lamin A/C, and LAP1 are highly relevant proteins for spermatogenesis, as reviewed in Pereira et al. [29], for which levels and distribution were evaluated by immunocytochemistry (Figure 7). Visible alterations of the basal levels and distribution of these proteins occurred after GC-1 cells exposure to 20 µg/mL of ZnO NPs. According to the present results, the levels of nesprin-1 (Figure 7A) did not change in the presence of ZnO NPs; however, SUN1 (* *p* ≤ 0.001) (Figure 7A) and LAP1 (* *p* < 0.0001) (Figure 7B) levels increased significantly in GC-1 cells exposed to ZnO NPs for 12 h. Further, lamin A/C increased significantly (* *p* < 0.0001) in GC-1 cells exposed to ZnO NPs for 6 h and 12 h (Figure 7B). In addition, it is important to note an increase in nesprin-1, SUN1, and LAP1 in areas of nucleus confinement and, on the contrary, lamin A/C decrease at these sites (Figure 7, arrows).

## 4. Discussion

The applications of ZnO NPs in biomedicine are numerous, given their multiple advantages conferred by the physicochemical properties of these specific nanomaterials. To date, only very few studies have explored the effects of ZnO NPs on the male reproductive system, and these are very important and should be explored in the future. We strongly believe that both in vitro and in vivo studies will contribute to determining the impact of ZnO NPs on male fertility. Previous studies have indicated that ZnO NPs have significative cytotoxic effects on spermatogenesis. ZnO NPs have been reported as a dose- and time-dependent cytotoxic inducer in testis and in male germ cells. ROS production and DNA damage have been described as driving forces that induce apoptosis and cell cycle arrest in cells treated with ZnO NPs [16,17,18,19]. These ZnO NPs cellular effects have consequential negative repercussions on the histological integrity of testis [20,21,22] and in sperm quality, as previously described [10,16,17,18,21,23]. However, to date, there are no studies on the consequences of ZnO NP exposure in the first stage of spermatogenesis.

In the present study, GC-1 spermatogonia cells were used as a cell model to investigate the impact of exposure to low and high ZnO NPs concentrations for short and long periods of incubation The results evidenced the cytotoxicity of ZnO NPs in GC-1 cells in a dose- and time-dependent manner. Viability evaluation was assessed regarding the metabolic activity (Figure 3A) and the membrane integrity (Figure 3B) of GC-1 after their exposure to ZnO NPs. Data revealed that 6 h with 10 µg/mL of ZnO NPs was enough to significantly decrease the metabolism of GC-1 cells but not sufficient to damage its membrane and consequent loss of integrity. However, the highest concentrations of ZnO NPs (20 µg/mL) reduce the metabolic activity of mouse spermatogonia cells and induce the loss of cell membrane integrity. Furthermore, it is important to note that, except for cells exposed to 20 µg/mL of ZnO NPs, GC-1 viability did not significantly decrease after 12 h of ZnO NPs exposure. These results indicate that ZnO NPs concentrations lower than 20 µg/mL were not enough to induce negative and permanent impact in spermatogonia cells once GC-1 can recover from toxic effects of ZnO NPs (Figure 3A,B). These results agree with the studies of apoptosis and necrosis performed (Figure 3C). At 6 h, the number of apoptotic and/or necrotic cells was not significant while the number of cells in necrosis (permeable to PI) was significantly higher after 12 h of exposure with higher ZnO concentration tested, indicating that these cells are suffering a cell death by necrosis (Figure 3C), with loss of membrane integrity [40,41]. The significant loss of cell viability reported by the trypan blue assay agrees with the cell death results from flow cytometry. Overall, ZnO NPs are a toxic nanomaterial to GC-1 cells in a dose- and exposure time-dependent manner, where only higher concentrations and long exposure tested induced cell death with loss of cell metabolism and membrane integrity.

The production of high levels of ROS induced serious alterations in spermatogonia cells by promoting biomolecular oxidation, causing DNA fragmentation and consequently apoptosis or necrosis [45,46]. In the study here described using GC-1 cells, ROS intracellular levels significantly increased after 6 h of exposure to the maximum concentration evaluated (20 µg/mL) and after 12 h of exposure to 5, 10, and 20 µg/mL. According to these results, ZnO NP exposure hypothetically raises the intracellular content of Zn^2+^, increasing ROS production and thus oxidative stress, which may induce DNA damage in GC-1 cells exposed to higher concentration of these NPs (Figure 4). These oxygen radicals could have originated at the nanoparticle surface as well as from biological substrates such as damaged mitochondria by inhibition of cellular respiration or by open the mitochondrial pore, releasing the cytochrome C that results in cell death [47].

DNA damage in GC-1 cells is enhanced in the presence of higher concentrations of ZnO NPs after 6 and 12 h of exposure. The increase in γ-H2AX phosphorylated at Ser139 (Figure 4B) by protein kinase ATM (mutated in ataxia-telangiectasia) leads to the recruitment of the mediator of DNA damage checkpoint protein 1 (MDC1) as a response to the formation of double strand breaks (DSB). Therefore, DNA damage response (DDR) leads to cell repair and chromatin decondensation, increasing the accessibility of DNA to transcription and repair and the activation of checkpoint proteins which arrest the cell cycle progression [43]. Therefore, y-H2AX (S139) levels fluctuate over time, increasing exponentially in the first few min after the DSB and decreasing after a few hours [48]. In the present study, DNA damage was evaluated after 6 and 12 h of exposure to ZnO NPs and the results clearly indicate that the amount of γ-H2AX (S139) significantly increases with higher ZnO NP concentration upon 12 h of exposure. It is important to note that additional studies for monitoring full DNA damage after short times of exposure to ZnO NPs are recommended and should be addressed in future studies.

Furthermore, it is important to mention that DNA damage and necrosis might occur as a consequence of high Zn^2+^ intracellular levels. Although in this study the Zn^2+^ release by ZnO NPs dissolution was not assessed, it is essential to report that previous studies refer to cell cytotoxicity as a consequence of Zn^2+^ homeostasis breakdown [49]. Several authors reported Zn^2+^ increase as a product from ZnO NPs dissolution in lysosomes after cell uptake [24,26,50,51] or by ZnO NPs dissolution in the extracellular environment, which can result in transport of the dissolved Zn^2+^ ions into the cell. The intracellular Zn^2+^ levels increased, exceeding the capacity of Zn^2+^ homeostatic system. As a result, toxic Zn^2+^ levels cause the mitochondrial membrane potential breakdown, inducing mitochondrial generation of ROS and DNA fragmentation, which activates caspases and leads to apoptosis. At even more increased Zn^2+^ concentration, cell necrosis is the dominant form of cell death [49,52].

ZnO NPs also influenced the dynamics and structure of the GC-1 cell line. The cytoskeleton is an interconnected network of intracellular filamentous composed of microtubules, intermediate filaments, and actin filaments crucial for cell structural and shape maintenance, movement, division, and function [53,54]. Tubulin and actin are components of the cellular cytoskeleton that assemble microtubules and actin filaments, respectively. Actin filaments are distributed near the cell membrane, while microtubules are located in the cytoplasm between the cell nucleus and cell membrane [54,55]. Microtubules and actin filaments are continuously undergoing polymerization and depolymerization processes that are exquisitely controlled by intracellular proteins [56,57]. Acetylated α-tubulin has a role in stabilizing the structure of all microtubules, protecting microtubules from disruption [56,58] and repairing the damage on microtubule [59]. Any interference with microtubule dynamics during cell division produces aberrant spindles leading to apoptosis or to unbalanced chromosome distribution in the daughter cells [24]. ZnO NPs disrupt the cytoskeleton architecture or cytoskeletal components in different cell types, although this dysregulation is also dependent on cell type. Beyond shape modifications in cell morphology during cellular stress responses, the cytoskeleton disruption also causes alterations in cell signalling under sub-toxic conditions, including during exposure to NPs in which cell viability is unmodified or marginally decreased. Alterations on cytoskeleton components should be investigated as predictors not only of cell shape modifications but also of cell physiology in exposed cells to NPs [60]. Once again, the cytoskeleton evaluation in spermatogonia cells was not previously analysed. In GC-1 cells, the increase of acetylated α-tubulin, a marker of microtubules stabilization, is visible after higher exposure to ZnO NPs. The increase of acetylated α-tubulin may occur for protecting the MT structure from damage induced by ZnO NPs as a recent study indicates [58]. Further, the dynamics of β-tubulin exposure also decreases after a long exposure to high concentrations of ZnO NPs, which indicates that the microtubules are conditioned and that the protein transport is compromised, altering the cellular dynamics (Figure 5A,B; Figure 6). Besides that, β-actin (Figure 5C) does not significantly change, but F-actin (Figure 6) is reduced after 12 h of exposure to ZnO NPs. Although, the immunocytochemistry analysis revealed an increase of β-tubulin and F-actin after 6 h of exposure (Figure 6). Tubulin and actin are two zinc-scavenging proteins that undergo structural changes upon Zn^2+^ binding [61,62]. According to a previous study, Zn^2+^ binds directly to tubulin, stimulating its assembly [62], and induces F-actin polymerization and aggregation [61], which is in concordance with the increase of β-tubulin and F-actin fluorescence by immunocytochemistry analysis, respectively. At 12 h, the decrease of F-actin and β-tubulin may be associated with increased ROS production and the consequent microfilaments and microtubules disruption/dysfunction [25,26]. Changes in the cytoskeleton in the presence of high concentrations and long exposure times to ZnO NPs also compromise the adhesion and epithelial shape of GC-1 cells (data not shown).

Besides causing cytoskeleton alterations, ZnO NPs were also reported as an inducer of nuclear enlargement, of chromatin compaction [25], of nuclear DNA leakage and breakage [18], and of nuclear fragmentation [47]. In agreement, the nucleus from GC-1 cells exposed to ZnO NPs presented significant visible morphological deformities (Figure 6D). On spermatogenesis, the dynamic of NE is in constant rearrangement, and any alteration on NE can lead to serious infertility phenotypes [29]. For the first time, the effects of ZnO NPs at the nucleoskeleton level (SUN1, nesprin-1, lamin A/C, and LAP1) in spermatogonia cells were evaluated (Figure 7).

The NE is a selective structural barrier composed of a pair of distinct membranes, the inner (INM) and outer nuclear membranes (ONM), separated by the perinuclear space; the nuclear lamina and the nuclear pore complexes (NPCs) together defined the barrier between the cytoplasm and nucleus [29,63]. SUN and KASH protein domains are type II integral membrane proteins embedded in the INM and ONM, respectively, which physically interact in the perinuclear space to form a physical connection of nucleoplasm and cytoskeleton, the LINC complex [64,65,66,67]. LINC complexes are critical for nuclear integrity and play fundamental roles in nuclear positioning, shaping, and movement [63,68], providing a mechanism for transmission of mechanical forces from the cytoskeleton into the nucleus, directly affecting chromatin compaction and organisation and thus gene expression [64,66,69]. The nuclear shape is determined by the cytoskeleton, the nuclear lamina, and the chromatin distribution and condensation [63].

LAP1 is a crucial protein for the maintenance of the NE architecture and regulation of the cell cycle [38,70]. In addition, lamins stabilize the nuclear membrane and, along with LAP1, organize the nucleus by localizing specific proteins responsible for chromatin organization, cell cycle control, and transcription regulation to the nuclear periphery. The failure to properly localize nuclear components as a result of defective nuclear transport has been directly associated with defects in chromatin organization and gene regulation [71]. These characteristics turn lamin A/C and LAP1 into important proteins for cellular dynamics during the spermatogenesis [30]. According to immunocytochemistry studies (Figure 7), after GC-1 cells exposure to ZnO NPs, the nucleoskeleton proteins were redistributed to nuclear confinement zones (higher mechanotransduction stress points) (Figure 7, arrows). These stress areas are characterized by defects in lamina organization and by a high intranuclear pressure from actin-based nucleus confinement that can lead to NE rupture [72,73,74,75], which is in accordance with the depletion of lamin (A/C) (Figure 7B) and with the redistribution of SUN1 and nesprin-1 (proteins from the LINC complex) and of LAP1 to areas of nuclear confinement or fragility (Figure 7A) in GC-1 cells exposed to ZnO NPs. At the same time, the levels of SUN1, LAP1, and lamin A/C increase significantly while nesprin-1 levels do not undergo any significant changes (Figure 7). In addition, the increase of lamin A/C levels occurs as a response from DNA damage and ROS level increase induced by ZnO NPs (Figure 4). Lamin A/C has a key role in promoting both DNA repair and in preventing DNA damage and in ROS modulation [76], which justify the increase of lamin A/C even in GC-1 cells exposure for 6 h to 20 µg/mL ZnO NPs.

The mechanism as to how ZnO NPs compromise the distribution and levels of SUN1, nesprin-1, LAP1, and lamin A/C is not yet clear, and further studies are needed to understand their increase and the process that induces the delocalization. However, it is important to mention that SUN1 and nesprin-1 distribution changes and lamin disruption after ZnO NP exposure can lead to serious alterations in nuclear movement and positioning and to increase in genome instability. The mouse spermatogonia cellular model, GC-1, belongs to a cellular stage prior to meiosis, and given the significant nuclear alterations observed upon high dose of ZnO NP exposure, we hypothesise that meiosis progression may be compromised, affecting spermatogenesis, which might originate alterations on male fertility. In fact, several previous studies indicated that nuclear alterations lead to crucial meiosis changes affecting spermatogenesis, originating male infertility [29,76,77,78,79]; however, the same should be explored using ZnO NPs. Therefore, the results here presented raised questions about the use of ZnO NPs for the most diverse applications, since it allowed us to understand the effect of ZnO NPs in reducing the metabolic cell activity, the loss of cell membrane integrity, the increase in production of ROS, the DNA damage, and the cell death of spermatogonia cells. In addition, it was possible to realise that the cytotoxic effect in spermatogonia cells induces significant changes in the cytoskeleton and nucleoskeleton, questioning the physiological role of zinc oxide in terms of their regulation. This very interesting issue should be addressed in future studies.

However, the exact cytotoxic mechanism is not yet consensual. Some studies suggest that surface reactivity is responsible for the spontaneous ROS generation in cells exposed to ZnO NPs [45,51,80]. Other studies reported Zn^2+^ release by the dissolution of ZnO NPs as the main underlying cause of cytotoxicity [24,26,50,51]. The solubility of ZnO NPs is highly variable, depending on cell type, exposure conditions, routes of administration, and method of synthesis. [81]. Since these results are not able to associate ROS production as a cause of cytotoxicity or just a consequence of the cytotoxicity induced by Zn^2+^ release, an extensive comparative study between ZnO NPs with different physicochemical properties will be required to assess the influence of its nature on toxicity. In fact, the physicochemical characteristics of ZnO NP condition, its toxicokinetic, and the routes of exposure can lead to new biological and unforeseen interactions [82,83]. 

In addition, the relative risk of ZnO NPs in GC-1 cells cannot be assessed just by looking at concentration and time of exposure. Also, it will be important in the near future to assess the accumulation of ZnO NPs in vivo in the testis at low doses and at reduced exposure times.

## 5. Conclusions

The present study describes for the first time the cytotoxic effects of ZnO NPs on spermatogonia cells (GC-1) in a time- and dose-dependent manner. Low levels of ZnO NPs during a short period of exposure do not cause spermatogonia cells alterations. The significant increase in cell death was observed with higher ZnO NPs concentrations at 6 and 12 h and is likely to occur due to the increase in ROS levels causing DNA damage (DSBs), leading to H2AX phosphorylation at Ser139 by ATM activating the DDR signaling path. However, DDR is not able or sufficient to repair the DNA damage caused by higher concentrations of ZnO NPs. Significant changes were also observed in the cytoskeleton and nucleoskeleton, leading us to hypothesize that the cellular effects of ZnO NPs exposure resulted from alterations of cytoskeleton and nucleoskeleton regulatory mechanisms. These mechanisms should be explored given their potential to cause adverse consequences on spermatogenesis, compromising male fertility. Further research is being carried out to understand the mechanism of action of these nanomaterials, leading to cytoskeleton and nucleoskeleton alterations. Further, it will be interesting to extend these assays to later stages of spermatogenesis.
